# Occupational risk factors among Iranian farmworkers: a review of the available evidence

**DOI:** 10.4178/epih.e2017027

**Published:** 2017-07-02

**Authors:** Mahin Ghafari, Zahra Cheraghi, Amin Doosti-Irani

**Affiliations:** 1 Department of Public Health, School of Health, Shahrekord University of Medical Sciences, Shahrekord, Iran; 2 Department of Epidemiology, School of Public Health, Hamadan University of Medical Sciences, Hamadan, Iran

**Keywords:** Occupational health, Risk factors, Farmworkers, Iran

## Abstract

Farming is one of the most important components of most economies. No comprehensive picture exists of the health status of Iranian farmers and the work-related hazards that affect them. We aimed to determine the gaps in the current knowledge regarding the occupational health of Iranian farmworkers. Electronic databases including Medline, Web of Science, Scopus, and Embase, as well as national databases including the Scientific Information Database, MagIran, and Barakat Knowledge System, were searched for articles published through March 2017. All epidemiologic studies regarding the occupational health of farmworkers in Iran were reviewed, regardless of their design, language, time of publication, and location. Of the 86 retrieved articles, 39 studies were ultimately analyzed. Most studies were conducted in Fars, Kerman, and Mazandaran provinces. According to the results of this review, chemical, physical, and biological hazards, along with work-related injuries, may be the main factors threatening the health of farmworkers. The unsafe use of pesticides was related to male infertility, eye and digestive complications, pesticide poisoning, pesticide absorption, hematological changes, non-Hodgkin lymphoma, and multiple myeloma. Chemical hazards (e.g., the unsafe use of pesticides), physical hazards, injuries, and biological hazards (e.g., work-related infectious diseases) threaten the health of Iranian farmworkers. Moreover, farmworkers lack adequate knowledge about the occupational hazards they face and the relevant risk factors.

## INTRODUCTION

Farming is one of the most important components of the economy in most countries. It exerts major effects on both public health and food safety. The agricultural workforce, consisting of over 1.1 billion individuals, is the largest workforce in the world. Moreover, the health of farmworkers has an important role in food provision [1]. Farming is known as a high-risk job in both developed and developing countries [1,2]. In addition to exposure to physical, chemical, and biological risk factors, work-related injuries and accidents can also threaten the health of farmworkers [3-5].

Agriculture plays an important economic role in the lives of people in many countries, including Iran [6]. In 2015, more than 18.0% of employees (approximately 4 million people) in Iran worked in the farming sector [7]. According to previous studies, occupational hazards such as musculoskeletal disorders [8] and injuries [9], pesticide poisoning, skin cancer, and infectious diseases threaten the health of Iranian farmworkers [10-12]. Unfortunately, there is no comprehensive occupational health monitoring system for farming-related diseases and injuries in Iran. Based on the available health indices, in 2010, only 32.0% of Iranian farmworkers were registered with the health system. Of these, 18.7% were covered by health care services and regular screening tests [13].

However, despite the magnitude of agricultural areas and the agricultural workforce in Iran, limited epidemiologic research has evaluated the health status and potential occupational hazards among farmworkers in different regions of the country. We aimed to review all the published studies in the field of the occupational risk factors for farmers and to determine the gaps in the current knowledge regarding the occupational health of Iranian farmworkers.

## MATERIALS AND METHODS

### Searching

Electronic databases, including Medline, Web of Science, Scopus, and Embase, were searched for studies published until March 2017. The search keywords were occupational injuries, occupational exposure, occupational diseases, occupational health, biohazard release, chemical hazard release, physical hazard, farmer, farmworkers, agricultural workers, and Iran. We also searched national databases, such as the Scientific Information Database, MagIran, and Barakat Knowledge System, for articles published until the same date. The reference lists of the selected studies were also evaluated.

All epidemiologic studies about the occupational health of Iranian farmworkers were included, regardless of their design, language, location, and time of publication. Case reports and letters to the editor were excluded.

### Study selection and data extraction

Two authors (Z Cheraghi and A Doosti-Irani) assessed the titles and abstracts of the retrieved articles. The full texts of the selected studies were then reviewed according to the eligibility criteria. The first author’s name, study design (cross-sectional, case-control, or cohort) and aims, sample size, year of publication, language, location of the study, the participants’ gender and mean age, type of occupational hazard, type of outcome or disease, and the main findings of the selected studies were extracted.

#### Quality assessment

Two authors (Z Cheraghi and A Doosti-Irani) were responsible for quality assessment. For cohort and case-control studies, the Newcastle-Ottawa Quality Assessment Scale was used for quality determination [14]. For cross-sectional studies, we used the modified version of the Newcastle-Ottawa scale. The maximum score on this scale is 8. We categorized the quality of studies based on this score as follows: scores of 6 and above indicated high quality, 5-6 moderate quality, and 4 and lower low quality.

Microsoft Excel (Microsoft Corp., Redmond, WA, USA) was used for the extraction and management of data. Descriptive data analysis was performed in order to obtain the frequency and distribution of conducted studies according to the type of occupational hazard studied, the type of outcome or occupational disease, and the location of the study. The ArcMap version 9.3 (Esri, Redlands, CA, USA) was used to generate a map of Iran based on published studies in the field of occupational health among farmworkers.

## RESULTS

A total of 86 articles, including 7 from Web of Science, 2 from PubMed, 46 from Scopus, and 31 from national databases, were selected. After removing the duplicates (n= 19), the titles and abstracts of the remaining 67 articles were evaluated. In the next step, the full texts of 45 articles were checked according to the eligibility criteria, and 39 studies [10,12,15-50] were finally included in this review.

**Figure 1.**
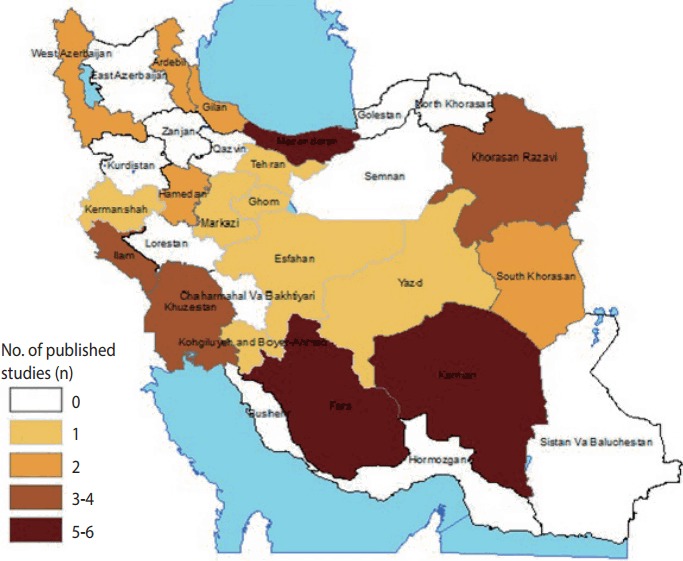
The distribution of the published studies regarding the occupational health of Iranian farmworkers.

The distribution of the selected studies across provinces is shown in Figure 1. No studies assessed the occupational health of farmers in east Azerbaijan, Zanjan, Kurdistan, Ghazvin, Golestan, and north Khorasan provinces. Most studies were conducted in Fars, Kerman, and Mazandaran provinces. The characteristics of the included studies are summarized in Table 1.

### Studies regarding chemical hazards

Twelve studies assessed chemical hazards, including pesticide exposure, among farmworkers. Neghab et al. [11] examined the relationship between the prevalence of infertility and pesticide exposure among farmworkers. They found infertility to be more prevalent among farmworkers than in the general population (p< 0.05) and suggested pesticide exposure as a possible cause.

Aghilinezhad et al. [16] assessed the effects of pesticides on the health of farmworkers. Based on their findings, 68% of the farmworkers did not use personal protective equipment during the administration of pesticides. In addition, eye and digestive complications of pesticide exposure were quite common among the studied farmworkers.

Mazloomi Mahmoodabad et al. [35] designed an interventional study to clarify the effectiveness of the health belief model (HBM) on the preventive behaviors of farmworkers regarding exposure to pesticides. They concluded that HBM-based education was effective in promoting preventive behaviors among farmworkers (p< 0.001).

According to Ayuzi & Poornajaf [19], 25.2% of the studied farmworkers reported occupational poisoning with pesticides. Ebrahimzadeh et al. [20] and Shayeghi & Shayeghi [44] assessed the absorption rate of pesticides among farmworkers in rice farms. They reported that the absorption of pesticides was higher among the farmworkers than in the general population.

Emam et al. [21] evaluated the effects of pesticides on hematological parameters among farmworkers. They found that hematological parameters such as hemoglobin (p= 0.002), hematocrit (p= 0.001), and prothrombin time (p= 0.001), were higher in farmworkers than in workers with other occupations. Pakravan et al. [39] measured plasma cholinesterase activity before and after exposure to organophosphate pesticides in farmworkers. Based on their findings, pesticide exposure decreased plasma cholinesterase levels by 50%.

Zakerinia et al. [49] found an association between pesticide exposure and both non-Hodgkin lymphoma (odds ratio [OR], 3.9; 95% confidence interval [CI], 2.2 to 6.8) and multiple myeloma (OR, 2.48; 95% CI, 1.16 to 5.2).

Hashemi et al. [26] and Hamerezaee et al. [24] studied the factors affecting the safe use of pesticides and knowledge regarding safety in the administration of pesticides, respectively. They both highlighted the necessity of training courses on the safe use of pesticides for farmworkers. In a cross-sectional study, Malekirad et al. [34] showed exposure to organophosphorus pesticides to be associated with neuropsychological disorders (p< 0.001).

### Studies regarding physical hazards

Eleven studies focused on physical hazards among farmworkers. Shirinkam & Fani [45] compared the outcomes of pregnancy among farmers and non-farmers. They reported a higher incidence of preterm delivery and low birth weight among female farmworkers, but the difference was not statistically significant.

Four studies assessed the role of sun protection in skin cancer prevention [15,37,43,47]. They all underscored the significance of training and educational interventions on sun-protecting behaviors in skin cancer prevention among farmworkers.

Tirgar et al. [48] measured the risk of heat disorders among farmworkers. They indicated that farmworkers were prone to a variety of heat exhaustion disorders. Moreover, farmworkers lacked adequate knowledge regarding the risk of heat exhaustion on their health.

Three cross-sectional studies [8,41,46] determined the prevalence of musculoskeletal disorders and related risk factors among farmworkers. They calculated the prevalence of musculoskeletal disorders in the elbows, knees, back, and legs to be 19.8, 52.0-58.3, 46.5, and 27.0%, respectively.

Two studies [18,50] focused on the noise pollution of agricultural machinery and identified noise pollution as an occupational hazard among the drivers of agricultural machinery. They thus recommended the use of hearing protective devices, improvements in the cabin of agricultural machinery, educational interventions, and hearing exams to be essential for the prevention of hearing disorders among farmworkers.

### Studies regarding injuries

Three studies focused on occupational injuries among farmworkers. According to Javadi & Rostami [32], three groups of factors (personal, mechanical, and environmental factors) affected the incidence of injuries. They reported that 53% of injuries were related to personal factors and that 40% were related to a combination of both mechanical and personal factors. Esmaeili et al. [22] found falls from trees (41.4%) to be the most common cause of work-related injuries among farmworkers. Moreover, the feet (35.7%) and hands (25.7%) were the most frequently damaged organs. Rafiei et al. [40] identified hand amputations as the most common injury (54%) among farmworkers.

### Studies regarding biological hazards

Six studies were related to biological hazards and work-related infectious diseases among farmworkers. Two studies assessed eye and respiratory allergies in saffron farmers [23] and respiratory problems among sheep farmers [25]. Studies on work-related infectious diseases have indicated that leptospirosis was common in rice farmers [17], avian influenza H9N2 was prevalent among poultry workers [27], and cryptosporidiosis [31] and brucellosis [10] were common among farmworkers in general.

Studies on the knowledge, attitude, and practices of farmers regarding occupational health.

Six studies [12,28-30,33,36] assessed the knowledge of farmworkers regarding their occupational health. They concluded that educational interventions were necessary for health promotion and work-related disease prevention among the farmworkers.

## DISCUSSION

Based on the reviewed articles, chemical, physical, and biological hazards, as well as occupational injuries, are the main threats to the health of Iranian farmworkers. However, since most studies were limited to particular provinces (such as Kerman, Fars, and Gilan) and no research in this field was conducted in a number of provinces, the available evidence cannot be interpreted as providing an accurate picture of the health status of the farmworkers throughout the country. However, based on the available health indicators in 2010, only 32.0% of Iranian farmworkers were registered with the health system and 18.7% of them were covered by occupational health care services [13].

According to the results of the reviewed studies, the unsafe use of pesticides causes male infertility, eye and digestive complications, pesticide poisoning, pesticide absorption, hematological changes, non-Hodgkin lymphoma, and multiple myeloma. Therefore, despite the abovementioned limitations, the available evidence confirms that the unsafe use of pesticides is a considerable threat to the health of Iranian farmworkers. Based on the results of studies in other countries, exposure to pesticides is associated with other diseases such as prostate cancer [51], Parkinson disease [52], breast cancer, thyroid cancer, and ovarian cancer [53]. Educational interventions about the safe use of pesticides and periodic checkups of farmworkers are hence necessary to prevent pesticide-related diseases among Iranian farmworkers.

Physical hazards such as heat, sunlight, and improper physical work also threaten the health of Iranian farmworkers. Farmworkers are at risk of skin cancer because of exposure to ultraviolet radiation [54]. Long working hours, especially in the summer, increase the risk of skin cancer among Iranian farmworkers. Moreover, Iranian farmworkers lack adequate knowledge regarding sun-protective behaviors [12,15]. Thus, educational interventions about skin cancer prevention are also essential for Iranian farmworkers. In addition, due to the inadequacy of the available evidence regarding skin cancer among Iranian farmworkers, further studies from all agricultural areas of Iran are required before preventive interventions are planned.

Based on the evaluated studies, musculoskeletal disorders are also prevalent in Iranian farmworkers. These disorders are generally common among all farmworkers [55,56]. Nevertheless, more studies are warranted to identify the prevalence and related risk factors of these disorders among Iranian farmworkers. Ergonomic and educational interventions are also necessary to encourage farmworkers to adopt a correct posture during physical work.

Occupational injuries are prevalent among Iranian farmworkers. Injuries to the hands, feet, and eyes can affect the health, quality of life, and economic status of farmworkers. Some injuries may even lead to death. Injuries thus impose a considerable burden on the families of farmworkers, the agricultural sector, and the community. Since only 3 studies assessed work-related injuries among Iranian farmworkers, further studies are needed in this field.

Work-related infectious diseases, such as brucellosis, avian influenza, leptospirosis, Q fever, and cryptosporidiosis, are prevalent among farmworkers [10,27,57,58]. However, due to the limited number of studies (n= 4) on infectious diseases among Iranian farmworkers, the available evidence cannot be interpreted as reflecting the actual status of these diseases, and further epidemiologic studies in this field are warranted.

This review had the limitation that most of the included studies were cross-sectional and therefore did not provide sufficient evidence regarding the risk factors of work-related diseases. Moreover, while the quality of some studies was low, we included all available studies, regardless of quality, due to the limited number of available studies.

Based on the results of this review, it is recommended that Iranian health policymakers and occupational health researchers develop a national registry system to register occupational diseases and injuries among farmworkers, pay more attention to the health status of farmworkers at the national and subnational levels, perform research on the occupational health of farmworkers, determine research priorities based on epidemiologic findings, and clarify the health status of farmworkers by designing a national cohort study.

## CONCLUSION

Although insufficient evidence exists regarding the health status of Iranian farmworkers, the available evidence showed that work-related diseases are prevalent in farmers. Chemical hazards (e.g., the unsafe use of pesticides), physical hazards, injuries, and biological hazards (e.g., work-related infectious diseases) threaten the health of Iranian farmworkers. Moreover, the knowledge of farmworkers about work-related hazards and risk factors is not sufficient.

## Figures and Tables

**Table 1.** Characteristics of the studies included in the review

First author (publication year)	Location	Quality	Sample size	Design	Hazard	Outcome	Main results
Neghab (2014) [11]	Fars	M	268	CS	Chemical: exposure to pesticides	Infertility	The prevalence of current primary infertility was 7.4%
Aghilinezhad (2008) [16]	Four province^1^	M	1,279	cs	Chemical: exposure to pesticides	Eye and digestive complications of pesticides	The prevalence of eye and digestive complications was 0.2-3.0%
Mazloomi Mahmoodabad (2016) [35]	Kerman	M	128	1	Chemical: exposure to pesticides	Improvements in practice	There was a significant increase in the mean scores of knowledge (p=0.002) and health belief model components (p=0.001), in addition, the mean score of preventive behaviors increased after intervention (p=0.001)
Ayuzi (2004) [19]	llam	M	550	cs	Chemical: exposure to pesticides	Occupational poisoning	25.2% of participants-claimed to have been poisoned and 10.0% expressed that some other members of their own families had been poisoned
Ebrahimzadeh (2005) [20]	Mazandaran	L	70	cs	Chemical: exposure to pesticides	AChE enzyme activity	AChE activity in the male workers was significantly lower than the control group (p<0.05) There was a significant difference between the mean of AChE activity in the female workers and the control group (p<0.05)
Shayeghi (2003) [44]	Mazandaran	L	35	cs	Chemical: exposure to pesticides	AChE enzyme activity	The amount of AChE activity was about 65.0% in 5.0% of the subjects, 75.0% in 20.0% of them, 87.5% in 40.0% of them and 100% in 35.0% of them
Emam (2012) [21]	Khuzestan	L	108	cs	Chemical: exposure to pesticides	Occupational poisoning	The values of Hb, Hct, RBC, platelets, and PT in the case group were greater than in the controls Hematological indices were different in the case group, but not meaningfully compared to the normal range (p〉0.05)
Pakravan (2016) [39]	Mazandaran	M	36	c	Chemical: exposure to pesticides	Plasma ChE activity	The plasma ChE level decreased after exposure by over 50%
Zakerinia (2012) [49]	Fars	L	400	cc	Chemical: exposure to pesticides	Lymphoid neoplasm	Out of the 200 cases that were diagnosed as lymphoid neoplasms, 100 were non-Hodgkin lymphoma, 54 Hodgkin lymphoma, and 46 multiple myeloma 72% of the non-Hodgkin lymphomas were of the B-cell type, 15% of theT-cell type, and the rest were not classified
Hashemi (2012) [26]	Fars	H	155	cs	Chemical: exposure to pesticides	Safe use of pesticides	The most important factors of the perceived importance and competence of farmers on the safety measures were the experience of pesticide-related adverse health effects in the past and the official education of farmers, respectively
Malekirad (2013) [34]	Southwest Iran	M	374	cs	Chemical: exposure to pesticides	Neurocognitive, menta health, and glucose disorders	The exposed farmers showed higher FBG (p<0.001), BUN (p<0.007), Cl (p<0.001), oral GTT (p<0.001), and lower AST (p<0.001), ALP (p<0.001), and creatinine (p=0.004) than controls
							The prevalence of anxiety/insomnia and severe depression was higher in the farmers than in controls (p<0.05 and p<0.001, respectively)
Hamerezaee (2016) [24]	West Azerbaijan	M	290	cs	Chemical: exposure to pesticides	Knowledge about the safety	98% of farmers believed that pesticides can have severe adverse effects on health, however 53.7% of them did not use personal protective equipment
Shirinkam (1999) [45]	Gilan	M	460	CS	Physical	Outcome of pregnancy	The incidence of postpartum uterine atony in farmer women was significantly higher than in housewives (p<0.05)
Tirgar(2012) [48]	Mazandaran	M	340	cs	Physical	Heat disorders	95% of the workers were not informed about pre-hydration in a hot environment and none of them know about the use of electrolytes
Afshari (2016) [15]	Hamadan	H	200	CS	Physical: sunlight	Skin cancer	Among farmers, 31.5, 53.5, 3.0, and 65.0% used sunscreen, hats, gloves, and protective gear, respectively, and 81.0% of farmers did not use eyeglasses
Morowatishari-fabad (2015) [37]	Fars	H	300	cs	Physical: sunlight	Skin cancer	The association between age, sex, education, income, family history of sunburn with protective behaviors was significant (p<0.05) A history of sunburn increased sun-protective behaviors by 2.05 times
Sadeghi (2014) [43]	Kerman	M	200	I	Physical: sunlight	Skin cancer	Perceived susceptibility, severity, benefits, barriers, cues to action, and self-efficacy increased significantly in the intervention group (p<0.001)
Jafari Roodbandi (2015) [8]	Kerman	M	350	cs	Physical	MSDs	The highest prevalence of MSDs was in the knees (58.30%) and the lowest in the elbows (8.19%)
Tazval (2016) [47]	Ham	H	246	cs	Physical	Skin cancer	The preventive behaviors for skin cancer were associated with perceived susceptibility, severity, response efficacy, and self-efficacy
Aliabadi (2012) [18]	Hamadan	M	NR	cs	Physical: noise	Noise pollution	The highest and the lowest noise level around the tractors were 83.8 dB(A) and 73.9 dB(A) for the John Deere and Romani tractors, respectively The effect of the tractor engine speeds on the noise level was statistically significant (p<0.01)
Zamanian (2012) [50]	Khuzestan	M	NR	cs	Physical: noise	Noise pollution	The difference between the sound inside and outside of the driver’s cabin was low
Razavi (2014) [41]	Khorasan Razavi	H	400	cs	Physical	MSDs	The prevalence of MSDs in one or both knees, the waist, and one or both feet and the ankle during one year was 52.0,46.5, and 27.0, respectively There was a significant association between wrist pain and individuals’weight (p<0.05), pain in one or both knees and age (p<0.001), pain in one or both knees and height (p<0.05) and pain in shoulders and age (p<0.05)
Taghavi (2017) [46]	Kohgiluyeh and Boyer-Ahmad	M	NR	cs	Physical	MSDs	The poorest risk scores were associated with the following tasks: (1) manure disposal, (2) filling feed bags, and (3) pouring milk into a bucket Other tasks such as filling corn containers, pouring corn into the milling machine, preparing the feed, pouring food into mangers, attaching the milking machine, and pouring milk from a bucket into a tank imposed high risk
Fereidouni (2005) [23]	Khorasan Razavi	L	167	cs	Biological	Eye and respiratory allergies	24% of saffron workers showed allergic symptoms in saffron picking season and 11% suffered from allergic symptoms in other seasons The clinical symptoms included sneezing, watery nose, itchy eyes, itchy nose, and red eyes
Alavi(2014) [17]	Khuzestan	H	288	cs	Biological	Leptospirosis	Of the total of 288 samples, 65 (22.5%) were positive for IgM anti-leptospira antibodies There was a significant difference between the case and control groups regarding leptospiral infection (p<0.001)
Hashemi (2006) [25]	Khorasan Razavi	M	173	cs	Biological	Respiratory problems	The proportions of sheep breeders with wheezing (16.5%), asthma (14.0%), cough (29.0%), breathlessness (31.5%), and flu-like illness (38.0%) were higher than in agricultural farmers
Heidari(2016) [27]	Fars	L	200	CS	Biological	Influenza	Two percent of the poultry workers were positive for the A/chicken/ lran/12VIR/9630/1998 virus
Izadi (2014) [31]	Isfahan	M	422	cs	Biological	Cryptosporidiosis	The prevalence of *Cryptosporidium (C) *was 8.5%. *C. parvum* was identified in 72% of the positive farm workers and 65% of the positive household members Contact with calves (p<0.0001) was an important risk factor of *C. parvum* infection A negative association was observed between *C parvum* infection and cleaning of shoes/boots after daily work (p=0.004), hand washing (p=0.013), and the use of piped water (p<0.006)
Mohammadkhani (2015) [10]	Kerman	H	187	CS	Biological	Brucellosis	The prevalence of brucellosis was 3.2% A history of brucellosis in the family, working in the veterinary network, and working in semi-industrial versus industrial dairy farms were the risk factors for the disease
Rafiei (2011) [40]	Ardebil	L	100	cs	Injuries	Hand injuries	Subjects (62.0%) worked more than 8 hr/d and 64.7% experienced severe injuries in their hands Amputation was the most common injury in the participants
Esmaeili (2009) [22]	Kerman	L	70	cs	Injuries	Injuries	Falling from palm trees was the most common mechanism of injury (41.4%) The legs and hands were the main injured areas (61.4%)
Javadi (2007) [32]	National	H		cs	Injuries	Safety assessments	Tractors and rotating parts were associated with the highest percentage of injuries in machine-related accidents. Insufficient levels of education and training were the main personal factors related to agricultural accidents
Moradhaseli (2014) [36]	Kermanshah	H	140	cs	NA	Knowledge and practice regarding occupational hazards	There was a significant difference between the pre-test and post-test in the examination group, showing the relative effectiveness of the educational course
Kara mi (2015) [34]	Khuzestan and llam	H	230	cs	NA	Knowledge about safety	Safety knowledge was at a suitable level (mean, 3.73 of 5.00; SD, 1.41) and 72.22% of them were assessed medium
Heidari (2007) [28]	Ghom	H	200	cs	NA	Knowledge and practices regarding occupational health	Farmers had a low level of knowledge (10%) regarding occupational respiratory, gastrointestinal and renal disorders The lowest level of knowledge was related to preventive behaviors, including not blowing the nozzle of the pesticide sprayer (18.0%), noise reduction measures (18.5%), and application of sunscreen (20.0%)
Hosseini (2011) [29]	South Kho-rasan	M	818	cs	NA	Knowledge and attitudes regarding occupational health	The mean score of knowledge for preventive behaviors was 14.62±2.07 out of 18, regarding the protective equipment was 4.94±1.78 out of 7, and the symptoms of poisoning was 7.9±4.02 out of 16 A significant relationship was found between the score of knowledge of preventive behaviors with education level (p=0.003), age (p=0.002) and location (p<0.001)
Babazadeh (2016) [12]	West Azerbaijan	M	238	cs	NA	Skin cancer	There was a significant association between perceived threat (p=0.001) and threat appraisal with education (p=0.040), while 50.04% of participants had a low threat appraisal to skin cancer Perceived severity was high only in 15.5% of the individuals
RostamAbadi (2014) [43]	Markazi	H	294	cs	NA	Work Ability Index	The mean Work Ability Index score was 35.1 (SD, 10.6) Work ability was more strongly associated with the physical scales of the health dimensions, such as physical function, role-physical, and general health, whereas a lower association was found for mental scales, such as mental health

AChE, acetylcholinesterase; Hb, hemoglobin; Hct, hematocrit; RBC, red blood cells; PT, prothrombin time; ChE, cholinesterase; FBG, fasting blood glucose; BUN, blood urea nitrogen; Cl, chloride; GTT, glucose tolerance test; AST, aspartate aminotransferase; ALP, alkaline phosphatase; MSD, musculoskeletal disorder; IgM, immunoglobulin M; SD, standard deviation; H, high; M, moderate; L, low; CS, cross-sectional; I, interventional; C, cohort; CC, case-control; dB, decibel.

1 Gilan, Mazandaran, Kerman, Tehran.
